# Sex-Specific SARS-CoV-2 Mortality: Among Hormone-Modulated ACE2 Expression, Risk of Venous Thromboembolism and Hypovitaminosis D

**DOI:** 10.3390/ijms21082948

**Published:** 2020-04-22

**Authors:** Sandro La Vignera, Rossella Cannarella, Rosita A. Condorelli, Francesco Torre, Antonio Aversa, Aldo E. Calogero

**Affiliations:** 1 Department of Clinical and Experimental Medicine, University of Catania, 95123 Catania, Italy; sandrolavignera@unict.it (S.L.V.); rossella.cannarella@phd.unict.it (R.C.); rosita.condorelli@unict.it (R.A.C.); francesco.torre@icloud.com (F.T.); acaloger@unict.it (A.E.C.); 2 Department of Experimental and Clinical Medicine, Magna Graecia University Catanzaro, 88100 Catanzaro, Italy

**Keywords:** SARS-CoV2, COVID-19, ACE2: venous thromboembolism, vitamin D deficiency, gender differences

## Abstract

Severe acute respiratory syndrome coronavirus (SARS-CoV-2) disease (COVID-19) appears to have a higher mortality rate in presence of comorbidities and in men. The latter suggests the presence of a possible sex-dependent susceptibility. An enzymatic system involved in this different predisposition could be represented by angiotensin converting enzyme 2 (ACE2). ACE2 is activated and down-regulated by the spike protein of the virus and allows the penetration of SARS-CoV-2 into epithelial cells and myocardium. Data on the experimental animal have shown that 17ß-estradiol increases the expression and activity of ACE2 in both adipose tissue and kidney. Spontaneously hypertensive male mice have a higher myocardial ACE2 expression than females and its levels decrease after orchiectomy. In addition to this first aspect, the recent evidence of an increased frequency of venous thromboembolism in patients with COVID-19 (a clinical element associated with a worse prognosis) calls the attention on the safety of treatment with testosterone, in particular in hypogonadal men with greater genetic predisposition. Evidence that sex hormones are able to modulate the expression of ACE2 could help in interpreting epidemiological results and in designing more appropriate intervention strategies. Moreover, the vitamin D deficiency in elderly men may be worthy of further study regarding the epidemiological aspects of this different susceptibility and lethality between sexes.

Severe acute respiratory syndrome coronavirus (SARS-CoV-2) infection disease (COVID-19) was firstly described in December 2019 in Wuhan, Hubei province, China and officially declared a Public Health Emergency by the WHO on January 30, 2020, having infected more than 100,000 people in over 100 countries [1]. Data published by the WHO on April 15, 2020 show a number of Coronavirus cases as high as 2,000,243, with 126,758 deaths (https://www.worldometers.info/coronavirus/). The study of COVID-19 epidemiology is important to understand the pathogenic mechanisms and to design better therapeutic strategies.

Current evidence has shown that the COVID-19 mortality rate is comorbidity-dependent. In 45,000 Chinese patients positive to COVID-19, the crude case mortality rate was 0.9% in patients without comorbidities and it increased to 10.5%, 7.3% and 6.3% in patients with cardiovascular disease (CVD), diabetes mellitus or hypertension, respectively [2]. Italy has recorded 162,488 confirmed cases and 21,067 deaths (April 15, 2020) (https://www.worldometers.info/coronavirus/). According to the last available age-related analysis of mortality, released by the Italian “Istituto Superiore di Sanità” (ISS) on March 30, 2020, 8.9% of the deceased patients were >90 years old, 39.7% were 80–89 years old, 35.5% were 70–79 years old, 11.2% were 60–69 years old and 0.9% were 40–49 years old. Similar to Chinese statistics, SARS-CoV-2 shows higher mortality rates in patients with one or more comorbidities. In Italy, only 2.1% of deaths were represented by people with no documented comorbidity, whereas 21.3%, 25.9% and 50.7% regarded patients with one, two, three or more comorbidities, respectively. A tentative hypothesis explaining the epidemiologic findings is the high expression of the angiotensin-converting enzyme 2 (ACE2), which is activated by the viral spike protein in alveolar epithelial cells and myocardium [3].

ACE2 is a membrane amino-peptide with the catalytic subunit pointed towards the extracellular space of pulmonary, cardiac, renal and intestinal tissues. It functions as an angiotensin II-degrading enzyme which generates angiotensin (1-7). This latter oligopeptide has vasodilatory, hypotensive and diuretic effects [4,5]. So far, the binding of COVID-19 spike protein to ACE2 has been shown to downregulate ACE2 and, in turn, to decrease angiotensin (1-7) production. This mechanism may be involved in the pathogenesis of pulmonary hypertension and insufficiency caused by SARS-CoV-2 infection [6]. Therefore, the different ACE2 expression occurring in patients with hypertension, CVD or diabetes should be taken into account when the different mortality rate is considered in patients with these comorbidities.

One of the most frequently reported epidemiologic data is sex-related COVID-19 mortality. The evidence supports a higher predominance of men in several countries; thus, the male sex has been considered a poor prognostic factor by some authors [7]. Male sex represents 73% of deaths in China [8], 59% in South Korea [9] and, in Italy, ISS reports a prevalence of 70% of men among deceased people. A recent scoping review on all the available epidemiological studies, collecting data from 59,254 patients from 11 different countries, has shown an association between male sex and higher mortality rate [10]. These findings suggest the presence of a male-related susceptibility. Indeed, Channappanavar and colleagues [11] have shown that male mice are more susceptible to SARS-CoV infection than age-matched females. This increased susceptibility was associated with high virus titers, increased vascular leakage and alveolar edema. These changes were accompanied by increased accumulation of inflammatory monocyte/macrophages and neutrophils in the lungs of male mice and the depletion of monocyte/macrophages partially protected these mice from lethal SARS. In addition, the sex-specific differences were independent of T and B cell responses, due to the sex-related intrinsic difference in innate immunity [12]. Finally, ovariectomy or treatment of female mice with an estrogen receptor antagonist increased the mortality rate; therefore, suggesting a protective effect for the estrogen receptor signaling pathway in SARS-CoV infected mice [11]. In this context, the analysis of a possible hormonal-dependency of the expression and/or activity of ACE2 in the various tissues is of relevance to understand the pathologic mechanisms behind the epidemiologic findings.

ACE2 is expressed in both mouse and human adult Leydig cells, but in a testosterone-independent manner. In these cells, the enzyme has been proposed to play a role in steroidogenesis [13]. ACE2 has also been described in ovarian granulosa cells and its expression increases with a rise in luteinizing hormone (LH). Indeed, stimulation with human chorionic gonadotropic (hCG) improves the ACE2-angiotensin (1-7)-Mas system [14]. This may represent a compensatory mechanism that could be useful for avoiding an increase in blood pressure in postmenopausal women [15]. In addition to gonadal expression, some studies have shown an expression and activity of ACE2 influenced by sex hormones in the mouse adipose tissue, kidneys and myocardium [15,16,17,18]. Gupte and colleagues studied the contribution of ACE2 in the pathogenesis of the sex-related difference of obesity-related hypertension. Male mice fed with a high-fat diet showed a decreased ACE2 expression in the kidney, resulting in reduced angiotensin (1-7) and increased angiotensin (1-8) levels. This caused blood hypertension that was reversed by the administration of losartan [14]. The same authors had previously shown a low expression of ACE2 in the adipose tissue of male mice [15]. In contrast, high-fat-fed female mice have an increased expression of ACE2 in the adipose tissue, which leads to high angiotensin (1-7) levels and no hypertension until angiotensin (1-7) receptor antagonists are administered. Ovariectomy results in a decrease ACE2 activity in the adipose tissue that leads to hypertension, which is restored by 17ß-estradiol administration [16]. In the myocardium, ACE and ACE2 expression, as well as cardiac hypertrophy, are significantly higher in spontaneously hypertensive male mice compared to female mice. After orchiectomy, a significant decrease in ACE, ACE2 expression and cardiac hypertrophy was found and, consequently an increased cardiac performance was observed. Finally, ovariectomy causes an increase in ACE2 expression and cardiac hypertrophy, which worsens the function of the heart pump [15].

Taken together, these findings suggest a role of sex hormones in the sex-related expression/function of ACE2. This may help the interpretation of epidemiologic data. More in detail, 17ß-estradiol may increase the expression/activity of ACE2 in the adipose tissue and the kidney. As reported above, spontaneously hypertensive male mice showed higher ACE2 expression than female mice [15], which may provide a possible explanation for the higher mortality in men due to more severe cardiovascular damage that the viral infection is capable of causing. Since the expression of ACE2 in the myocardium appears to be modulated by androgen [15], a role for androgen receptor (AR) gene polymorphisms cannot be excluded in the pathogenesis of cardiovascular adverse events and hypertension in COVID-19-positive male patients.

Another aspect that needs to be highlighted is the increased CVD risk in patients with COVID-19. The prevalence of venous thromboembolism (VTE) in these patients has been reported as 25% and VTE is associated with an unfavorable prognosis [19]. Anticoagulant therapy, mainly with low molecular weight heparin, appears to be associated with a better prognosis in severe COVID-19 patients who have sepsis-induced coagulopathy criteria or markedly elevated D-dimer levels [20]. The scientific debate regarding the risk of VTE in patients treated with testosterone is very current. In a recent case-crossover study, 39,622 men were enrolled and 3110 of them (7.8%) had hypogonadism. In age-adjusted models, testosterone replacement therapy was associated with a higher risk of VTE in men with (odds ratio 2.32) and without (odds ratio 2.02) hypogonadism [21]. Among the various causes of hypogonadism, greater attention should be paid to those forms associated with a greater risk of VTE, such as in patients with Klinefelter syndrome [22]. The use of estrogen in women could expose them to the same risk, although in these cases the increased awareness of the risk of developing VTE is often preceded by appropriate screening.

Another aspect that may explain sex-related susceptibility may relate to vitamin D. Vitamin D deficiency has been shown to be independently associated with increased risk of viral acute respiratory infection (ARI) in a number of observational studies. A meta-analysis of clinical trials on vitamin D supplementation to prevent ARI onset has shown moderate protective effects [23]. Respiratory monocytes/macrophages and epithelial cells constitutively express the vitamin D receptor (VDR) thereby protecting against respiratory infections. Interestingly, 1,25OH_2_-Vitamin D3 and other VDR agonists lower significantly the pro-inflammatory response to antigen challenge in cystic fibrosis airway epithelial cells in vitro. Some studies suggest that low vitamin D levels may increase the risk or severity of respiratory viral infections, and interventional studies have shown that low levels are associated with increased expression and secretion of pro-inflammatory cytokines and chemokines. Interestingly, vitamin D administration decreases the inflammatory response to viral infections in airway epithelium without jeopardizing viral clearance. This suggests that adequate vitamin D levels would contribute to reduced inflammation and less severe disease in respiratory syncytial virus-infected individuals [24].

It has been known for many years that vitamin D deficiency is not adequately evaluated in older men. Deficiency [25(OH)D <20 ng/ml] has been estimated in 26%, and insufficiency (<30 ng/ml) in 72%. In particular, the deficiency is common among men during the winter and spring (especially in the northern communities) and in the oldest and more obese men [25]. Therefore, we speculate that one of the factors involved in this sex-related different susceptibility may due to less vitamin supplementation in men in their sixties compared with age-matched women.

In conclusion, the role of gonadal hormones and possible replacement therapy should be reconsidered in both sexes at this historic moment of SARS-CoV-2 pandemic. Testosterone (or LH/hCG) administration could be temporarily discontinued or given at a lower posology in patients with hypogonadism. In contrast, estrogen replacement therapy may be fully considered in hypogonadal and postmenopausal women. Furthermore, the involvement of the polymorphisms of AR activator and repressor proteins [26] as well as the role of selective androgen receptor modulators [27] as therapeutic agents in men could be deserving of investigation. However, this relationship is much more complex than it might seem at first glance. Indeed, it should be taken into account that the viral infection itself worsens the function of Leydig cells, as recently shown in [28], and the consequent hypogonadism exposes the patient to an increased cardiovascular risk and a decreased vitamin D level [29,30]. Close monitoring of serum testosterone levels in these patients would, therefore, be helpful and should also be continued when the acute phase of the disease has ended. It would also be interesting to know the length of active disease for SARS-CoV-2 patients with or without hypogonadism, since hypogonadism can worsen the systemic inflammatory response, as demonstrated on peripheral blood leukocytes where testosterone inhibits immune stimuli–induced secretion of proinflammatory cytokines, such as TNF and IFNγ [31]. Finally, vitamin D deficiency, regardless of testosterone levels, in elderly men may be worthy of further epidemiological evaluation to better understand the different susceptibility and lethality between sexes.
